# Pretreatment prognostic nutritional index and immune status for response to preoperative neoadjuvant chemotherapy in cervical squamous cell carcinoma: an exploratory retrospective study

**DOI:** 10.3389/fonc.2026.1929427

**Published:** 2026-08-21

**Authors:** Zhou-Le Li, Jian-Bin He, Xue Liu, Hui-Ting Hao, Han-Xiao Zhu, Min Wang, Xiao-Ming Wu, Chun-Qiang Lu

**Affiliations:** 1 Department of Radiology, The Jinhua Affiliated Hospital of Wenzhou Medical University, Jinhua, China; 2 Nurturing Center of Jiangsu Province for State Laboratory of AI Imaging & Interventional Radiology, Department of Radiology, Zhongda Hospital, School of Medicine, Southeast University, Nanjing, China

**Keywords:** cervical squamous cell carcinoma, immunonutritional status, magnetic resonance imaging, preoperative neoadjuvant chemotherapy, prognostic nutritional index

## Abstract

**Introduction:**

Pretreatment nutritional and immune status may be associated with response to preoperative neoadjuvant chemotherapy (NACT) in cervical squamous cell carcinoma (CSCC), but its clinical utility remains uncertain.

**Methods:**

This exploratory retrospective study evaluated the association of pretreatment immunonutritional and tumor-related factors with short-term radiologic response to NACT. Ninety-two patients with histopathologically confirmed CSCC who received paclitaxel plus platinum-based NACT followed by surgery were included. Response was defined as a ≥30% reduction in the sum of target-lesion diameters on post-NACT magnetic resonance imaging relative to pretreatment imaging.

**Results:**

Sixty patients were responders and 32 were non-responders. Responders had higher pretreatment prognostic nutritional index (PNI) values, and FIGO stage distribution differed between groups. Higher PNI remained associated with response after adjustment for FIGO stage and pretreatment maximum tumor diameter (odds ratio [OR] = 1.154, 95% confidence interval [CI] 1.030-1.295; *P* = 0.014) and after adjustment for pretreatment MRI-assessed lymph-node metastasis and maximum tumor diameter (OR = 1.136, 95% CI 1.023-1.262; *P* = 0.017), with consistent findings in the weighted sensitivity analysis. Repeated nested cross-validation yielded areas under the receiver operating characteristic curve of 0.630 for PNI alone, 0.684 for five-variable ridge logistic regression, and 0.737 for random forest. Neither multivariable model showed a statistically reliable improvement over PNI alone. The random forest showed a train-validation AUC gap of 0.163 and was considered unstable and exploratory.

**Discussion:**

Pretreatment PNI may provide a simple marker for preliminary response risk stratification, but prospective external validation is required before clinical application.

## Introduction

Cervical cancer remains one of the leading causes of cancer-related mortality among women worldwide, and cervical squamous cell carcinoma (CSCC) is the most common histological subtype (1, 2). Globally, cervical cancer continues to impose a substantial disease burden (3). Preoperative neoadjuvant chemotherapy (NACT) followed by surgery has been explored in selected patients with CSCC, particularly when tumor shrinkage may improve surgical feasibility or provide early information on chemosensitivity (4, 5). However, the role of NACT varies across disease stage, tumor burden, and institutional practice, and it should not be interpreted as a universally indicated strategy for all early-stage patients. Non-responders may be exposed to treatment-related toxicity and potential delay of definitive therapy (6, 7). Therefore, clinically accessible markers that help estimate short-term radiologic response before NACT may be useful for exploratory risk stratification, while their ability to guide treatment decisions requires further validation.

CSCC is a heterogeneous malignancy, and response to NACT may be influenced by tumor biology and host-related factors, including pretreatment immunonutritional status (8, 9). Adequate nutritional reserve is important for maintaining treatment tolerance, reducing chemotherapy-related complications, and supporting completion of planned therapy (10, 11). Serum albumin reflects nutritional reserve and systemic inflammatory burden, whereas lymphocytes are central to antitumor immune competence; therefore, indices integrating these components may provide clinically accessible information on host status (12, 13). Pelvic MRI is routinely used for local staging and treatment-response assessment in cervical cancer, and MRI-derived tumor features have also been investigated for pretreatment prediction of NACT response (14, 15).

In addition to nutritional status, systemic inflammation and immune function may also influence chemotherapy response in patients with CSCC by shaping immune surveillance, inflammatory activity, and the tumor microenvironment (16, 17). Lymphocytes are central to antitumor immune responses, whereas neutrophils, platelets, and monocytes are closely involved in systemic inflammation and tumor progression (17). Accordingly, several composite hematological indicators, including the neutrophil-to-lymphocyte ratio (NLR), platelet-to-lymphocyte ratio (PLR), and monocyte-to-lymphocyte ratio (MLR), have been investigated as prognostic or predictive markers in patients with cancer, including those receiving chemotherapy (18–20). The prognostic nutritional index (PNI), calculated from serum albumin level and peripheral lymphocyte count, integrates nutritional and immune components and may provide a comprehensive reflection of the patient’s pretreatment immunonutritional status (18, 21). Although previous studies have investigated the prognostic significance of nutritional and immune status in patients with cervical cancer, most have focused on long-term survival outcomes (21–23). Short-term radiologic response provides an earlier measure of treatment sensitivity than survival endpoints and may facilitate timely multidisciplinary reassessment when response is inadequate (7, 15). Compared with radiomics-based prediction models, PNI is derived from routinely available laboratory tests and does not require image segmentation, feature extraction, or specialized computational pipelines, which may improve its clinical accessibility (21, 24, 25). Further investigation is therefore needed to clarify whether pretreatment immunonutritional indicators are associated with radiologic response to preoperative NACT in patients with CSCC.

This study aimed to evaluate whether pretreatment nutritional and immune status, together with composite hematological indicators and tumor-related parameters, was associated with short-term radiologic response to preoperative NACT in patients with CSCC. The study was intended to identify candidate markers for future validation rather than establish a clinical decision rule. As a secondary exploratory objective, we compared PNI alone with multivariable ridge logistic regression and random forest to determine whether integrating routinely available clinical variables provided additional discriminatory value beyond PNI alone.

## Materials and methods

### Study subjects

**Figure 1:**
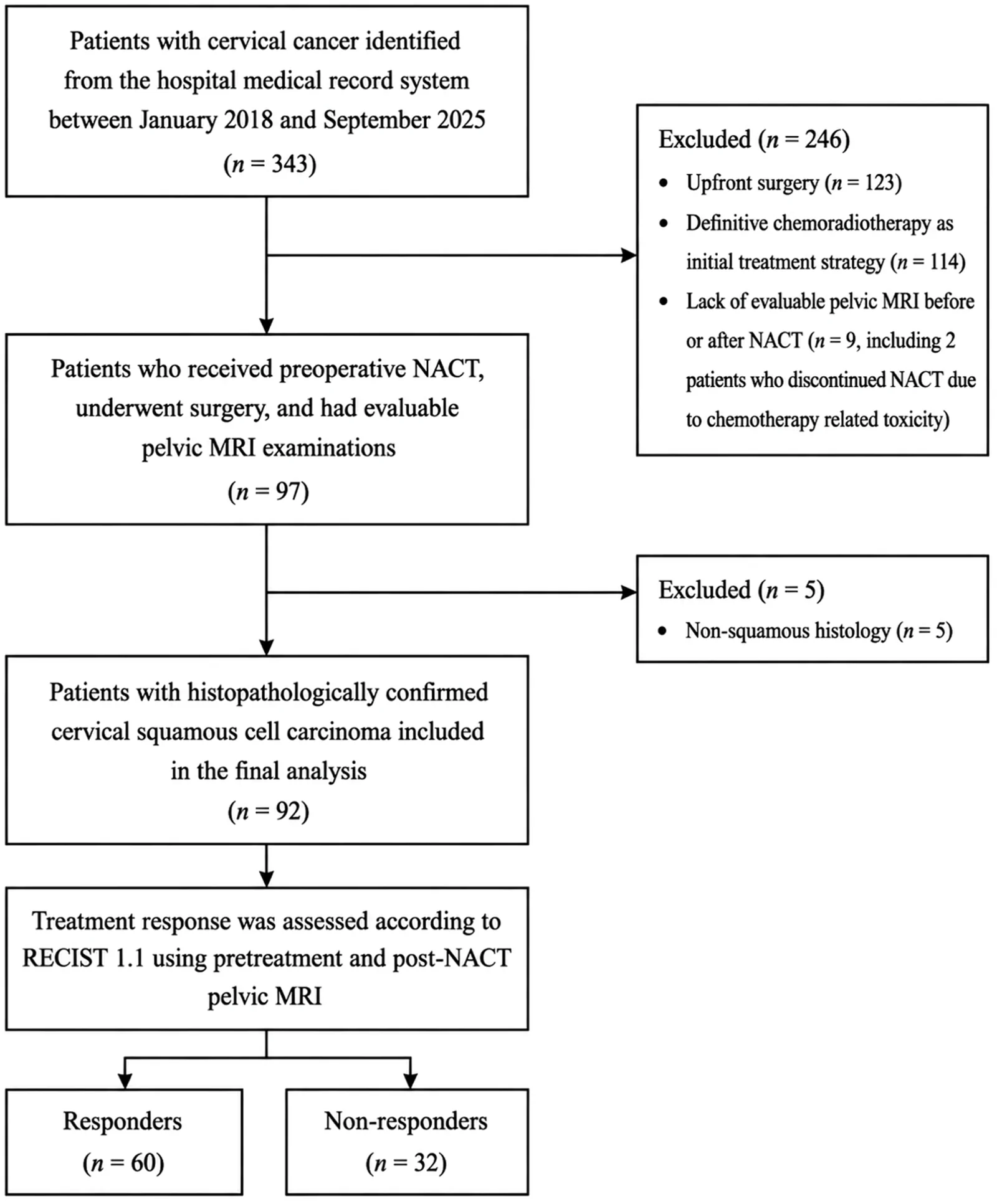
Flow chart of patient selection and response classification after neoadjuvant chemotherapy. NACT, neoadjuvant chemotherapy; MRI, magnetic resonance imaging.

Patients with histopathologically confirmed CSCC who initiated preoperative NACT at our institution between January 2018 and September 2025 were retrospectively reviewed. Patients were included in the radiologic response analysis if they were aged ≥18 years, had stage IB2–IVA disease according to the 2018 International Federation of Gynecology and Obstetrics (FIGO) staging system, had received no anti-tumor treatment before preoperative NACT, completed at least two cycles of NACT, had evaluable pretreatment and post-NACT magnetic resonance imaging (MRI) examinations, subsequently underwent surgery, and had complete clinicopathological records required for the planned analyses. The treatment strategy was determined through multidisciplinary team evaluation, taking into account tumor stage, tumor burden, pretreatment MRI-assessed lymph node metastasis (LNM), surgical feasibility, and the patient’s overall condition. For patients with FIGO stage III–IVA disease, preoperative NACT was considered only when no distant metastasis was identified and tumor downstaging was expected to permit complete resection. Surgery was subsequently performed when post-NACT reassessment confirmed resectability. All patients underwent radical hysterectomy combined with bilateral pelvic lymphadenectomy. Radical hysterectomy consisted of removal of the uterus and cervix together with bilateral parametrial tissues and the upper vagina. The exact extent of parametrial and vaginal resection was determined by the operating surgeon according to residual tumor extent and intraoperative findings. The NACT regimen consisted of paclitaxel combined with a platinum agent every 3 weeks for 2–3 cycles before surgery (26). Paclitaxel was generally administered at 135–175 mg/m². The platinum component included cisplatin at 60–75 mg/m², nedaplatin at 80 mg/m², or carboplatin, with the carboplatin dose calculated according to renal function as follows: dose (mg) = 5 × (glomerular filtration rate [GFR] + 25). The choice of platinum agent was individualized according to renal function, hydration tolerance, comorbidities, and anticipated toxicity (27). Patients were excluded if they had a history of other malignancies or conditions that could affect hematological or biochemical parameters, including acute or chronic infection, hematologic disorders, or the use of medications that might influence blood test results before treatment. Patients who did not complete at least two cycles of NACT or did not have an evaluable post-NACT MRI examination were also excluded from the response analysis. This included two patients who discontinued NACT before completing two cycles because of chemotherapy-related toxicity and subsequently received definitive concurrent chemoradiotherapy; neither had an evaluable post-NACT MRI examination within the predefined response-assessment interval. The patient-selection process is summarized in **Figure 1**.

### Clinical and pathological data collection and index calculation

Demographic characteristics, tumor-related variables, treatment details, and laboratory test results were obtained from hospital medical records. Postoperative lymphovascular space invasion, stromal invasion depth, and tumor differentiation were extracted from the corresponding surgical pathology reports. These variables were analyzed as supportive postoperative pathological characteristics rather than as measures of pathological response. Although all patients in the final cohort underwent surgery, a uniform pathological complete-response or tumor-regression grading system was not prespecified or consistently documented and therefore could not be reliably reconstructed retrospectively. Patients without an available HPV test result were classified as ‘Unknown’ and retained as a separate descriptive category without imputation. HPV status was not included in the regression or prediction models. Hematological indicators measured within 7 days before the initiation of NACT were collected for analysis. The nutritional and immune-related indicators based on hematological parameters were calculated as follows: prognostic nutritional index (PNI) = serum albumin (g/L) + 5 × lymphocyte count (10^9/L); neutrophil-to-lymphocyte ratio (NLR) = neutrophil count/lymphocyte count; monocyte-to-lymphocyte ratio (MLR) = monocyte count/lymphocyte count; platelet-to-lymphocyte ratio (PLR) = platelet count/lymphocyte count; eosinophil-to-lymphocyte ratio (ELR) = eosinophil count/lymphocyte count; and fibrinogen-to-albumin ratio (FAR) = fibrinogen level (g/L)/albumin level (g/L) (23).

### MRI acquisition and image analysis

Pelvic MRI examinations were performed using either a 3.0-T MAGNETOM Vida system (Siemens Healthineers, Erlangen, Germany) or a 3.0-T Ingenia system (Philips Healthcare, Best, the Netherlands), with a multichannel phased-array body coil. The routine protocol included multiplanar T2-weighted imaging, axial diffusion-weighted imaging (DWI), apparent diffusion coefficient (ADC) maps, and contrast-enhanced fat-suppressed T1-weighted imaging. DWI was acquired with a high b value of 1000 s/mm², and ADC maps were generated automatically on the corresponding MRI workstation. The principal scanner-specific acquisition parameters are summarized in **Supplementary Table 1**. Pretreatment MRI was performed before the first NACT cycle, whereas post-NACT MRI was performed within 7 days after completion of the final NACT cycle and before surgery.

Pretreatment and post-NACT pelvic MRI images were independently reviewed by two radiologists, each with 10 years of experience. The radiologists were blinded to clinical outcomes and any previously assigned treatment-response classification during image review. Imaging variables included tumor size in three orthogonal dimensions (transverse, anteroposterior, and craniocaudal), apparent diffusion coefficient values, lymph node status, and tumor necrosis. For each patient, pretreatment maximum tumor diameter was defined as the largest of these three measurements. On post-NACT MRI, the same lesion was measured on the corresponding or closest comparable plane and orientation. Pretreatment MRI-assessed LNM was determined from pelvic MRI performed before the initiation of NACT. For ADC measurement, the primary cervical tumor was identified on the axial high-b-value DWI and ADC maps with reference to the corresponding axial T2-weighted and contrast-enhanced T1-weighted images. On the axial slice showing the largest cross-sectional area of the tumor, each radiologist manually placed a freehand region of interest along the visible margin of the solid tumor component. Areas of macroscopic necrosis, cystic degeneration, hemorrhage, the cervical canal, and obvious imaging artifacts were excluded from the region of interest. ADCmean and ADCmax were automatically obtained from all voxels within the region of interest and were expressed as ×10^−^³ mm²/s.

Primary-tumor diameters and measurable lymph nodal short-axis diameters were independently measured on pretreatment and post-NACT MRI by the two radiologists, and ADC values were independently measured on the corresponding ADC maps. For each target lesion, the mean of the two radiologists’ measurements was used for subsequent quantitative analyses and for calculating the pretreatment and post-NACT sums of target-lesion diameters. Disagreements regarding target-lesion selection or matching of the same lesion between pretreatment and post-NACT MRI were resolved through discussion and consensus before the final measurements were determined. Inter-reader reliability for continuous measurements was assessed using intraclass correlation coefficients.

### Treatment-response assessment

Treatment response was assessed using a Response Evaluation Criteria in Solid Tumors (RECIST) 1.1-based target-lesion approach (28, 29). Each patient in this cohort had one measurable primary cervical lesion. The sum of target-lesion diameters comprised the longest diameter of the primary cervical lesion and the short-axis diameters of up to two measurable lymph nodes, when present. For the primary cervical lesion, the longest diameter was defined as the largest of the transverse, anteroposterior, and craniocaudal diameters measured on pretreatment MRI. Lymph nodes were considered measurable when their short-axis diameter was ≥15 mm on pretreatment axial MRI. When more than two measurable lymph nodes were present, up to two representative and reproducibly measurable lymph nodes were selected as target lesions.

The same primary cervical lesion and target lymph nodes were identified on post-NACT MRI using anatomical landmarks and measured on the corresponding or closest comparable plane and orientation. The pretreatment and post-NACT sums of target-lesion diameters were calculated using the mean measurements of the two radiologists. The percentage reduction in the sum of target-lesion diameters was determined relative to the pretreatment sum. Patients with a ≥30% reduction in the sum of target-lesion diameters, without new lesions or unequivocal progression of non-target disease, were classified as responders; all others were classified as non-responders. The radiologists did not independently assign responder/non-responder categories. The final response classification used in all analyses was calculated from the mean measurements of the two radiologists according to the prespecified response criterion; therefore, categorical inter-reader agreement was not assessed.

### Statistical analysis

#### Descriptive and comparative analyses

Analyses were performed in Python 3.13.9 using scikit-learn 1.7.2, SHAP 0.50.0, NumPy 2.3.5, pandas 2.3.3, SciPy 1.16.3, and statsmodels 0.14.5. Continuous variables were summarized as mean ± standard deviation or median (interquartile range) and compared using Welch’s t test or the Mann-Whitney U test, as appropriate. Categorical variables were summarized as numbers and percentages and compared using Pearson’s chi-square test, Fisher’s exact test, or a Monte Carlo chi-square test when expected cell counts were small.

Binary logistic regression was used to evaluate the association between pretreatment PNI and radiologic response to NACT. Covariates were selected *a priori* according to clinical relevance rather than univariable P-value screening. Because pretreatment MRI-assessed lymph-node metastasis (LNM) contributes to the 2018 FIGO staging system, FIGO stage and LNM were entered into separate models. Model 1 included PNI, categorical FIGO stage, and pretreatment maximum tumor diameter, whereas Model 2 included PNI, pretreatment MRI-assessed LNM, and pretreatment maximum tumor diameter.

As a sensitivity analysis, propensity scores for response-group membership were estimated using logistic regression with categorical FIGO stage, pretreatment MRI-assessed LNM, and pretreatment maximum tumor diameter as covariates. PNI was not included in the propensity-score model because its association with radiologic response was the primary estimand of interest. Inverse-probability weights were calculated from the estimated probability of each patient’s observed response status and applied to balance the measured covariates between responders and non-responders. Covariate balance before and after weighting was assessed using absolute standardized mean differences, with values <0.10 considered indicative of adequate balance. A weighted binary logistic-regression model including pretreatment PNI and pretreatment maximum tumor diameter was subsequently fitted to evaluate the association between PNI and radiologic response.

A conventional five-variable logistic-regression model included PNI, log_2_-transformed SCC-Ag, log_2_-transformed NLR, pretreatment MRI-assessed LNM, and categorical FIGO stage. FIGO stage was represented by two indicator variables, with IB2–IIA as the reference category. Given six predictor degrees of freedom and 32 patients in the smaller outcome group, this analysis was considered exploratory. SCC-Ag and NLR were additionally entered on their original scales in a sensitivity analysis. The association between categorical FIGO stage and pretreatment MRI-assessed LNM was quantified using Cramér’s V. Multicollinearity in the encoded predictor matrix used by the logistic-regression models was assessed using variance inflation factors after log_2_ transformation of SCC-Ag and NLR and dummy-variable encoding of FIGO stage, with IB2–IIA as the reference category.

Three prespecified prediction models were compared using identical data partitions: PNI-only logistic regression, five-variable ridge logistic regression, and random forest. The two multivariable models included PNI, log_2_-transformed SCC-Ag, log_2_-transformed NLR, pretreatment MRI-assessed LNM, and categorical FIGO stage. Performance was estimated using repeated stratified nested cross-validation with five outer folds repeated 20 times and four inner folds for hyperparameter tuning. All preprocessing and tuning were restricted to the corresponding training folds, and each patient’s outer-fold probabilities were averaged across repetitions.

Pairwise AUC differences were evaluated using 1,000 paired stratified out-of-bag bootstrap samples. The PNI-only and ridge logistic-regression models were refitted within each bootstrap sample, whereas the random forest used the modal configuration selected across the repeated nested-validation folds.

Performance was assessed using AUC, Brier score, calibration intercept and slope, accuracy, sensitivity, specificity, positive predictive value, negative predictive value, and F1 score. Classification metrics used a probability threshold of 0.5. Random-forest stability was assessed using a learning curve, 500 outcome permutations, and the mean train–validation AUC gap; a gap >0.10 was considered indicative of instability. SHAP values were calculated only for patients in the outer validation folds and were used descriptively.

Associations between PNI and the evaluated variables were assessed using Pearson, Spearman, point-biserial, or rank-biserial correlations, as appropriate to variable type and distribution. Patients with unknown HPV status were excluded from the HPV analysis. *P* values were adjusted across all 15 tests using the Benjamini–Hochberg false-discovery-rate procedure. All tests were two-sided, with *P* < 0.05 considered statistically significant.

## Results

### Patient characteristics

**Figure 2:**
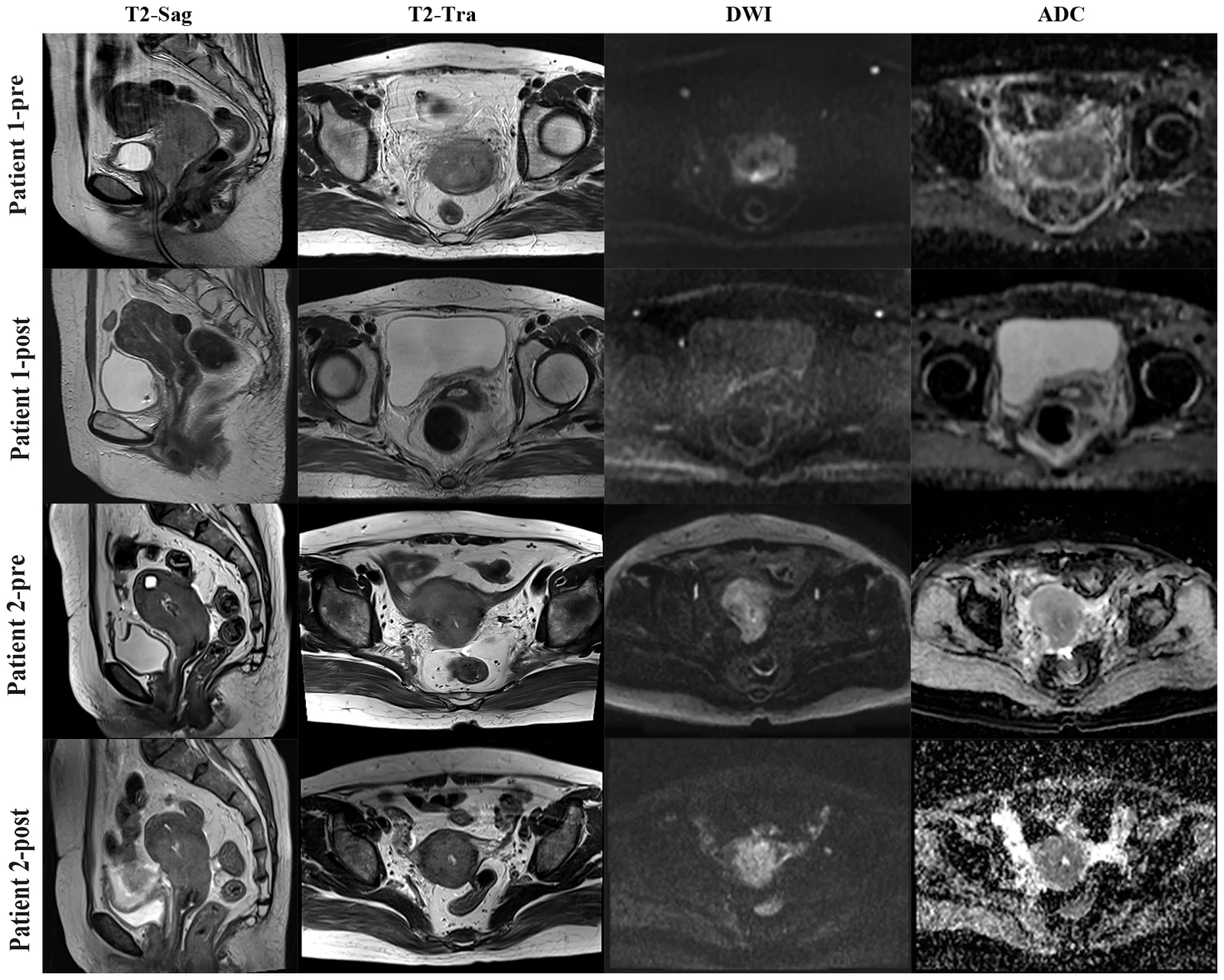
Pelvic MRI examples of chemotherapy responders and non-responders before and after preoperative neoadjuvant chemotherapy.

A total of 343 patients with cervical cancer were initially identified. Patients were excluded if they underwent upfront surgery (n=123), received definitive chemoradiotherapy as the initial treatment strategy (n=114), or did not have evaluable pretreatment or post-NACT pelvic MRI examinations (n=9). Among these nine patients without evaluable response MRI, two discontinued NACT before completing two cycles because of chemotherapy-related toxicity and subsequently received definitive concurrent chemoradiotherapy. Five additional patients with non-squamous histology were excluded. The final cohort comprised 92 patients with CSCC, including 60 responders and 32 non-responders. Representative pretreatment and post-NACT MRI findings in responders and non-responders are shown in **Figure 2**.

All patients had one measurable primary cervical lesion. Among the 37 patients with pretreatment MRI-assessed LNM, four had measurable lymph nodes that were included in the sum of target-lesion diameters. Responders had higher pretreatment PNI values than non-responders (49.4 [45.9–54.0] vs 47.5 [45.8–49.3], P = 0.026), and the distribution of FIGO 2018 stage differed between the groups (*P* = 0.011). Pretreatment MRI-assessed LNM was observed in 26 responders (43.3%) and 11 non-responders (34.4%), with no significant between-group difference (*P* = 0.541). Apart from PNI and FIGO stage, no significant between-group differences were observed in the other clinical, laboratory, pretreatment MRI-derived, treatment-related, or postoperative pathological characteristics (**Tables 1**, **2**). Specifically, postoperative lymphovascular space invasion, stromal invasion depth, and tumor differentiation did not differ significantly between radiologic responders and non-responders (all *P*>0.05; **Table 1**). These variables were evaluated descriptively and were not regarded as standardized measures of pathological response. Among the three patients with FIGO stage IVA disease, two underwent surgery after substantial tumor regression following NACT, whereas the third underwent surgery after multidisciplinary reassessment confirmed resectability. All patients underwent radical hysterectomy combined with bilateral pelvic lymphadenectomy, while the exact extent of parametrial and upper vaginal resection was individualized according to residual tumor extent and intraoperative findings. The ICCs for pretreatment and post-NACT maximum primary-tumor diameter measurements were 0.88 and 0.87, respectively. The corresponding ICCs for pretreatment and post-NACT measurable nodal short-axis diameter measurements were 0.87 and 0.86, respectively.

**Table 1:** Clinical and postoperative pathological characteristics by radiologic response.

Variables	Responders (n=60)	Non-responders (n=32)	*P*
Age (years)	58.5 (52.0–67.0)	54.5 (46.0–62.0)	0.117
BMI (kg/m²)	23.40 (21.18–26.15)	23.95 (21.71–26.79)	0.486
Menopause status, n (%)			
Yes	41 (68.3)	21 (65.6)	0.792
No	19 (31.7)	11 (34.4)	
HPV status, n (%)			
Yes	45 (75.0)	23 (71.9)	0.846
No	3 (5.0)	1 (3.1)	
Unknown	12 (20.0)	8 (25.0)	
SCC-Ag (ng/mL)	8.12 (2.30–23.12)	7.95 (5.29–20.66)	0.709
Pretreatment clinical FIGO 2018 stage, n (%)			
IB2–IIA	19 (31.67)	15 (46.88)	**0.011**
IIB	3 (5.00)	7 (21.88)	
III–IVA	38 (63.33)	10 (31.25)	
LVSI, n (%)			
Yes	38 (63.33)	17 (53.13)	0.342
No	22 (36.67)	15 (46.88)	
Stromal invasion depth, n (%)			
≤1/3	11 (18.33)	8 (25.00)	0.717
1/3–2/3	14 (23.33)	6 (18.75)	
≥2/3	35 (58.33)	18 (56.25)	
Tumor differentiation, n (%)			
G1	5 (8.33)	1 (3.13)	0.720
G2	40 (66.67)	22 (68.75)	
G3	15 (25.00)	9 (28.13)	
Platinum agent, n (%)			
Carboplatin	19 (31.67)	10 (31.25)	0.884
Nedaplatin	25 (41.67)	12 (37.50)	
Cisplatin	16 (26.67)	10 (31.25)	

BMI, body mass index; SCC-Ag, squamous cell carcinoma antigen; HPV, human papillomavirus; FIGO, International Federation of Gynecology and Obstetrics; NACT, neoadjuvant chemotherapy. LVSI, stromal invasion depth, and tumor differentiation were postoperative pathological characteristics and were analyzed descriptively; they were not used to define radiologic response or a standardized pathological-response endpoint. Bold values indicate statistical significance at P < 0.05.

**Table 2:** Pretreatment MRI-derived and immunonutritional characteristics by radiologic response.

Variables	Responders (n=60)	Non-responders (n=32)	*P*
Tumor size parameters on pretreatment MRI			
Transverse diameter (mm)	40.0 (33.8–52.2)	40.5 (36.0–50.0)	0.931
Anteroposterior diameter (mm)	36.0 (29.8–45.0)	37.5 (32.8–40.0)	0.730
Craniocaudal diameter (mm)	44.0 (30.8–50.2)	37.0 (31.5–42.0)	0.162
Pretreatment MRI-assessed LNM, n (%)			
No	34 (56.7)	21 (65.6)	0.541
Yes	26 (43.3)	11 (34.4)	
Diffusion characteristics			
ADCmean (×10^−^³ mm²/s)	788.0 (656.8–852.2)	782.5 (723.2–867.0)	0.408
ADCmax (×10^−^³ mm²/s)	939.0 (800.2–1028.0)	921.0 (846.8–1007.2)	0.734
Tumor necrosis on MRI, n (%)			
No	34 (56.7)	14 (43.8)	0.336
Yes	26 (43.3)	18 (56.3)	
PNI	49.4 (45.9–54.0)	47.5 (45.8–49.3)	**0.026**
NLR	2.75 (2.07–4.03)	3.19 (2.21–4.93)	0.256
MLR	0.20 (0.15–0.30)	0.22 (0.15–0.38)	0.354
PLR	144.5 (123.6–182.5)	150.3 (130.2–241.3)	0.125
ELR	0.057 (0.024–0.097)	0.072 (0.040–0.123)	0.133
FAR	0.076 (0.067–0.086)	0.078 (0.069–0.098)	0.577

ADC values were derived from diffusion-weighted imaging. PNI, prognostic nutritional index; NLR, neutrophil-to-lymphocyte ratio; MLR, monocyte-to-lymphocyte ratio; PLR, platelet-to-lymphocyte ratio; ELR, eosinophil-to-lymphocyte ratio; FAR, fibrinogen-to-albumin ratio; MRI, magnetic resonance imaging; ADC, apparent diffusion coefficient. Bold values indicate statistical significance at P < 0.05.

### Association between pretreatment PNI and radiologic response

In Model 1, higher pretreatment PNI remained associated with greater odds of radiologic response after adjustment for FIGO stage and pretreatment maximum tumor diameter (OR = 1.154 per one-unit increase, 95% CI 1.030–1.295; *P* = 0.014). The overall FIGO-stage term did not reach statistical significance (*P* = 0.051), and pretreatment maximum tumor diameter was not associated with response (OR = 1.009, 95% CI 0.973–1.045; *P* = 0.639).

In Model 2, higher pretreatment PNI remained associated with radiologic response after adjustment for pretreatment MRI-assessed LNM and pretreatment maximum tumor diameter (OR = 1.136, 95% CI 1.023–1.262; *P* = 0.017). Neither pretreatment MRI-assessed LNM nor pretreatment maximum tumor diameter was significantly associated with response (**Table 3**).

**Table 3:** Multivariable logistic regression analysis of pretreatment factors associated with response to neoadjuvant chemotherapy.

Model 1: Adjusted for FIGO stage and pretreatment maximum tumor diameter					
Variables	β	SE	P value	OR	95% CI
PNI	0.143	0.059	0.014	1.154	1.030–1.295
FIGO stage			0.051		
IB2–IIA	Reference			1.000	
IIB	-0.734	0.803	0.361	0.480	0.099–2.317
III–IVA	1.394	0.563	0.013	4.030	1.338–12.136
Pretreatment maximum tumor diameter (mm)	0.009	0.018	0.639	1.009	0.973–1.045
Model 2: Adjusted for pretreatment MRI-assessed LNM and pretreatment maximum tumor diameter					
Variables	β	SE	P value	OR	95% CI
PNI	0.128	0.054	0.017	1.136	1.023–1.262
Pretreatment MRI-assessed LNM	-0.178	0.460	0.700	0.837	0.340–2.063
Pretreatment maximum tumor diameter (mm)	0.024	0.017	0.154	1.025	0.991–1.059

OR, odds ratio; CI, confidence interval; SE, standard error; PNI, prognostic nutritional index; FIGO, International Federation of Gynecology and Obstetrics; MRI, magnetic resonance imaging; LNM, lymph node metastasis; NACT, neoadjuvant chemotherapy.

### Propensity score-weighted sensitivity analysis

After inverse probability weighting, the absolute standardized mean differences for FIGO stage, pretreatment MRI-assessed LNM, and pretreatment maximum tumor diameter were 0.006, 0.030, and 0.024, respectively, indicating adequate balance (**Supplementary Table 2**). In the weighted logistic-regression analysis, higher pretreatment PNI remained associated with radiologic response (OR = 1.120, 95% CI 1.043–1.202; *P* = 0.002), whereas pretreatment maximum tumor diameter was not significant (OR = 1.006, 95% CI 0.986–1.027; *P* = 0.539) (**Supplementary Table 3**).

### Complementary five-variable logistic regression

In the complementary conventional logistic-regression model, higher pretreatment PNI was associated with radiologic response (OR = 1.132, 95% CI 1.008–1.272; *P* = 0.036). Log_2_-transformed SCC-Ag and NLR, pretreatment MRI-assessed LNM, and categorical FIGO stage were not statistically significant. Given the limited sample size and wide confidence intervals, these estimates were interpreted cautiously (**Table 4**). Categorical FIGO stage and pretreatment MRI-assessed LNM showed a weak association (Cramér’s V = 0.141; χ²=1.831, df=2, P = 0.400). VIFs for the encoded predictor matrix ranged from 1.029 to 1.237, indicating no evidence of problematic multicollinearity (**Supplementary Table 6**).

**Table 4:** Five-variable logistic regression for radiologic response.

Variable	β	SE	Z	P	OR	95% CI
PNI (per unit)	0.124	0.059	2.097	0.036	1.132	1.008–1.272
SCC-Ag (per doubling)	-0.024	0.137	-0.176	0.861	0.976	0.746–1.278
NLR (per doubling)	-0.438	0.310	-1.411	0.158	0.646	0.351–1.186
Pretreatment MRI-assessed LNM (yes vs no)	0.950	0.776	1.224	0.221	2.587	0.565–11.846
FIGO: IIB vs IB2–IIA	-0.572	0.831	-0.688	0.491	0.565	0.111–2.877
FIGO: III–IVA vs IB2–IIA	0.999	0.714	1.398	0.162	2.715	0.669–11.010

The outcome was response=1. SCC-Ag and NLR were log2 transformed; their ORs represent the effect of a doubling. FIGO was treated as a nominal categorical variable with stage IB2–IIA as the reference category. The model had six predictor degrees of freedom; with 32 non-response events, this corresponded to 5.3 non-response events per predictor degree of freedom. SE, standard error; OR, odds ratio; PNI, prognostic nutritional index; SCC-Ag, squamous cell carcinoma antigen; NLR, neutrophil-to-lymphocyte ratio; LNM, lymph node metastasis.

### Exploratory predictive model performance

**Figure 3:**
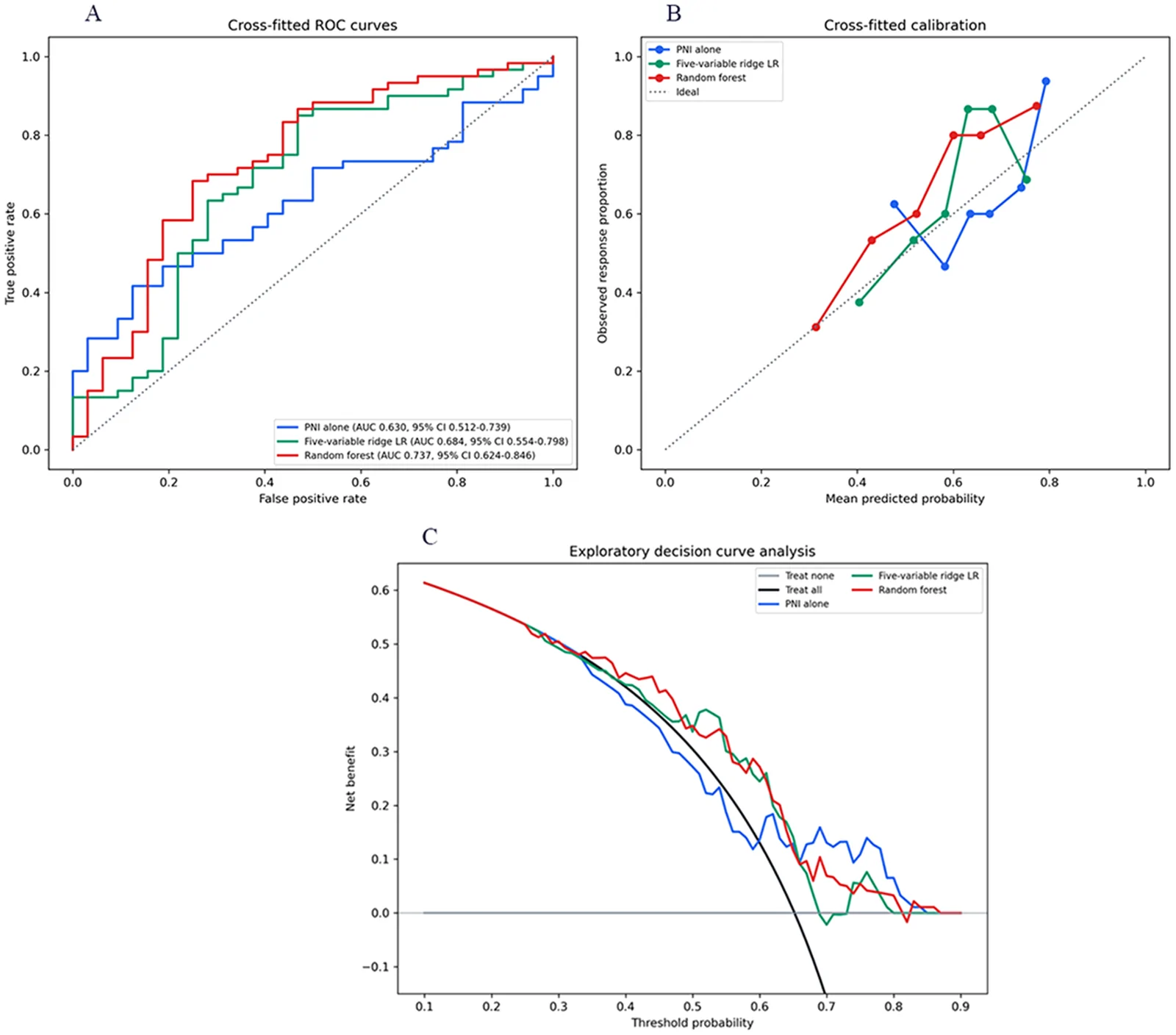
Cross-fitted model evaluation. **(A)** Receiver operating characteristic curves for PNI alone, five-variable ridge logistic regression, and random forest. **(B)** Calibration curves based on averaged outer-fold predictions. **(C)** Exploratory decision-curve analysis. The decision curves were not used to claim clinical utility because no clinically validated decision threshold was prespecified.

Repeated nested cross-validation produced AUCs of 0.630 (95% CI 0.512–0.739) for PNI alone, 0.684 (95% CI 0.554–0.798) for five-variable ridge logistic regression, and 0.737 (95% CI 0.624–0.846) for random forest (**Figure 3A**). The clinical ridge sensitivity model yielded an AUC of 0.690 (95% CI 0.567–0.811). Complete discrimination, calibration, and classification results are presented in **Supplementary Table 4**, and the calibration curves are shown in **Figure 3B**.

Paired bootstrap AUC differences were 0.051 for ridge logistic regression versus PNI (95% CI −0.135 to 0.193; *P* = 0.501), 0.082 for random forest versus PNI (95% CI −0.125 to 0.260; *P* = 0.378), and 0.031 for random forest versus ridge logistic regression (95% CI −0.099 to 0.156; *P* = 0.637) (**Supplementary Table 5**). Thus, neither multivariable model demonstrated a statistically reliable improvement over PNI alone. Results were similar when SCC-Ag and NLR were entered on their original scales. In this sensitivity analysis, higher PNI remained associated with radiologic response (OR = 1.145, 95% CI 1.016–1.291; *P* = 0.027), whereas SCC-Ag, NLR, pretreatment MRI-assessed LNM, and FIGO stage remained non-significant (**Supplementary Table 7**).

At the 0.5 threshold, the random forest yielded an accuracy of 69.6%, sensitivity of 75.0%, specificity of 59.4%, positive predictive value of 77.6%, negative predictive value of 55.9%, and F1 score of 0.763. The random-forest search space and hyperparameter-selection frequencies are reported in **Supplementary Table 8**.

### Random-forest stability and exploratory interpretation

The outcome-permutation test yielded an empirical *P* value of 0.006. The observed AUC of 0.691 in the fixed-configuration permutation pipeline was distinct from the primary nested cross-validated AUC of 0.737. Its mean training AUC of 0.902 exceeded the mean validation AUC of 0.739 by 0.163; therefore, the model exceeded the prespecified instability threshold and was considered unstable and exploratory (**Supplementary Figures 1**, **2**).

Exploratory decision-curve analysis did not demonstrate consistent net-benefit dominance of either multivariable model across the evaluated threshold range (**Figure 3C**). Because no clinically validated decision threshold was prespecified, this finding was interpreted descriptively.

Cross-fitted SHAP analysis ranked PNI as the feature with the highest mean absolute importance. However, because the random forest was unstable, the SHAP results were interpreted only as descriptions of model behavior, without directional, causal, or clinical interpretation (**Supplementary Figures 3**, **4**).

### Exploratory correlation analysis

Pretreatment PNI was weakly associated with pretreatment maximum tumor diameter (ρ = −0.223, *P* = 0.033) and FIGO stage (ρ = 0.217, *P* = 0.038), but neither association remained significant after FDR correction (both FDR-adjusted *P* = 0.201). None of the other evaluated associations between PNI and clinical, laboratory, pathological, or pretreatment MRI-derived variables remained significant after FDR correction (**Supplementary Table 9**).

## Discussion

In this exploratory retrospective cohort, higher pretreatment PNI was associated with short-term radiologic response to preoperative NACT in patients with CSCC. The direction of this association was consistent across the two prespecified adjustment models, the inverse probability-weighted sensitivity analysis, and the complementary five-variable logistic regression. Although the random forest yielded the numerically highest AUC, neither it nor the five-variable ridge logistic regression demonstrated statistically reliable incremental discrimination over PNI alone. The random forest additionally showed limited stability. Nevertheless, the discriminatory performance of PNI alone was modest; therefore, these findings remain hypothesis-generating and do not establish equivalence among the models or validate a clinical prediction rule.

PNI integrates serum albumin and lymphocyte count and has been investigated in several malignancies, including cervical cancer. These two components reflect overlapping aspects of nutritional reserve, systemic inflammatory status, and immune competence (21, 30–34). A low PNI may reflect limited nutritional reserve, systemic inflammation, or impaired immune competence, which may be associated with reduced chemotherapy tolerance or response (18, 35). In the present study, PNI remained associated with response in the five-variable model, but the lower confidence limit was close to the null and the model had six predictor degrees of freedom with only 32 patients in the smaller outcome group. Therefore, the precision and stability of this estimate remain limited. Pretreatment PNI showed weak unadjusted associations with maximum tumor diameter and FIGO stage, but neither remained statistically significant after FDR correction. These correlation findings should therefore be regarded as descriptive rather than mechanistic or causal. Selection bias and residual confounding also remain possible. Previous studies have reported associations between higher PNI and improved chemotherapy response or survival outcomes in several tumor types (36, 37). In cervical cancer, most existing studies have focused on the prognostic value of nutritional and immune indicators in relation to long-term survival (38, 39). Our study adds exploratory evidence focused on short-term radiologic response to preoperative NACT in CSCC. Recent evidence suggested that pretreatment immunonutritional status, assessed using the hemoglobin–albumin–lymphocyte–platelet score, was associated with lymph-node metastasis in cervical cancer. However, the endpoint of that study was nodal metastasis rather than response to NACT and therefore does not directly validate the present response findings (40).

FIGO stage distribution differed between responders and non-responders, whereas pretreatment MRI-assessed LNM did not. In Model 1, the overall FIGO-stage term did not reach statistical significance (*P* = 0.051), although the III–IVA versus IB2–IIA contrast was statistically significant. Given the small subgroup counts, wide confidence interval, heterogeneous treatment indications, and potential confounding by indication, this isolated contrast should not be interpreted as evidence that advanced disease is inherently more chemosensitive or that FIGO stage is a robust independent predictor of radiologic response. Pretreatment MRI-assessed LNM, SCC-Ag, and NLR were not statistically significant in their corresponding adjusted analyses. These estimates should be interpreted cautiously because of the limited sample size and wide confidence intervals. The cohort encompassed FIGO stages IB2–IVA and therefore represented heterogeneous disease stages and treatment indications. NACT followed by surgery was not applied uniformly across this stage range but was selected through multidisciplinary assessment, particularly for advanced-stage patients in whom tumor downstaging was expected to permit complete resection. Differences in tumor burden, surgical feasibility, treatment selection, and institutional practice may have introduced confounding by indication and affected the internal comparison of radiologic response. Although FIGO stage and pretreatment tumor burden were considered in the adjusted and weighted analyses, residual confounding cannot be excluded.

The model-validation results illustrate why the random forest should remain exploratory. Hyperparameter selection varied across folds, the learning curve showed weak performance at small training sizes, and the mean train–validation AUC gap was 0.163. The significant permutation result suggested that the model captured some outcome-related signal, but it did not establish reliable generalization or clinical benefit. Paired bootstrap intervals for all AUC differences crossed zero, and exploratory decision curves did not establish consistent net benefit. Consequently, the complex model was not treated as a clinical prediction tool, and SHAP directions were not emphasized. Repeated nested cross-validation was used to separate hyperparameter tuning from model-performance estimation and reduce optimistic bias (41). Cross-fitted SHAP values were interpreted only as descriptions of model behavior rather than independent biological or causal effects (42, 43). Nested validation requires model selection and tuning to be repeated inside the validation process rather than performed once using the complete dataset.

Although the random forest yielded the numerically highest AUC, discrimination alone is insufficient to establish clinical utility. Decision-curve analysis evaluates net benefit across threshold probabilities, but no clinically validated threshold was prespecified in this study. Because no clinically validated decision threshold was prespecified and the exploratory decision curves did not demonstrate consistent model dominance, these findings should be interpreted conservatively. Prediction-model assessment should consider calibration and clinical consequences in addition to AUC (44–47).

Several limitations should be noted. First, this was a single-center retrospective study with 92 patients and only 32 non-responders; therefore, some estimates remained imprecise and may be unstable. Second, the primary endpoint was short-term MRI response rather than standardized pathological response or long-term clinical outcomes. Although postoperative lymphovascular space invasion, stromal invasion depth, and tumor differentiation were reported descriptively, a uniform pathological complete-response or tumor-regression grading system could not be reliably reconstructed. Third, the cohort encompassed FIGO stages IB2–IVA and included only patients selected through multidisciplinary assessment for NACT followed by surgery who completed at least two cycles and had evaluable paired MRI. This clinical heterogeneity and indication-related selection may have affected internal validity and may limit generalizability, particularly to unselected patients with stage III–IVA disease or those receiving definitive chemoradiotherapy. Fourth, no independent external cohort was available; despite repeated nested cross-validation, bootstrap comparisons, learning-curve analysis, and permutation testing, the prediction models remain exploratory. Finally, residual confounding cannot be excluded despite multivariable adjustment and inverse probability weighting. Prospective multicenter studies using standardized pathological response and other clinically meaningful outcomes are required.

In conclusion, pretreatment PNI was associated with short-term radiologic response to preoperative NACT in this exploratory CSCC cohort. Neither multivariable model demonstrated statistically reliable incremental discrimination over PNI alone, and the random forest showed limited stability. These findings support further evaluation of PNI as a simple candidate marker for preliminary risk stratification but remain hypothesis-generating. Prospective multicenter validation against standardized pathological response and other clinically meaningful outcomes is required before clinical application.

## Data Availability

The original contributions presented in the study are included in the article/**Supplementary Material**. Further inquiries can be directed to the corresponding authors.
