# Supplementary material for: Pretreatment prognostic nutritional index and immune status for response to preoperative neoadjuvant chemotherapy in cervical squamous cell carcinoma: an exploratory retrospective study
Source: Front Oncol. 2026 Aug 21;16:1929427. doi: 10.3389/fonc.2026.1929427 (PMC13541484; doi:10.3389/fonc.2026.1929427)
Supplement: Supplementary file 1 [file SupplementaryFile1.docx]

Supplementary Materials

**Pretreatment prognostic nutritional index and immune status for response to preoperative neoadjuvant chemotherapy in cervical squamous cell carcinoma: an exploratory retrospective study**

# This file contains Supplementary Methods, Supplementary Tables S1–S9, and Supplementary Figures S1–S4.

# Supplementary Methods

## S1. Data audit and analysis dataset

All reanalyses used the end worksheet of the audited master workbook as the sole patient-level data source. The analysis dataset contained 92 patients, including 60 responders and 32 non-responders, with no missing values in the prespecified analysis variables. Response was coded as 1 and non-response as 0. The erroneous legacy PNI value of 134.4 was replaced with the verified master-record value of 54.3; the final observed PNI range was 37.95–58.05. Legacy stored prediction probabilities were not used. NACT cycle count and gluteal muscle area were removed because reliable patient-level values could not be reconstructed for the audited cohort.

## S2. Variable coding and transformations

FIGO stage was harmonized to three groups: IB2–IIA, IIB, and III–IV. Logistic-regression models treated FIGO as nominal, using IB2–IIA as the reference category and separate indicators for IIB and III–IV; no ordinal linearity assumption was imposed. For random forest, the same categories were encoded as 1, 2, and 3 to permit threshold-based tree splits. Platinum agent was coded as carboplatin, nedaplatin, or cisplatin. SCC-Ag and NLR were log2-transformed for the primary regression analyses because of right-skewed distributions; an original-scale sensitivity analysis is provided in Supplementary Table S6.

## S3. Prespecified models and nested cross-validation

Three prediction models were compared using identical outer data partitions: PNI-only logistic regression; five-variable ridge logistic regression containing PNI, log2(SCC-Ag), log2(NLR), LNM, and categorical FIGO stage; and random forest using the same five predictors. A clinical ridge sensitivity model included PNI, age, BMI, baseline maximum tumor diameter, FIGO stage, LNM, and platinum agent. Performance was estimated using stratified five-fold outer cross-validation repeated 20 times. Ridge penalties were chosen by four-fold inner cross-validation from C=10^-3 to 10^3. Within each outer fold, six reproducibly sampled random-forest configurations were evaluated from the prespecified 108-combination search space: n_estimators 300 or 800; max_features sqrt or 0.5; min_samples_leaf 5, 10, or 15; min_samples_split 10, 20, or 30; max_depth 3, 5, or unrestricted; and class_weight balanced. All preprocessing, encoding, standardization, tuning, and fitting were restricted to training data. Repeated out-of-fold probabilities were averaged at the patient level.

## S4. Performance, calibration, and paired model comparison

Patient-level averaged out-of-fold probabilities were used to calculate AUC, Brier score, calibration intercept, calibration slope, accuracy, sensitivity, specificity, positive predictive value, negative predictive value, and F1 score. Classification metrics used a fixed threshold of 0.5. AUC confidence intervals were obtained by stratified bootstrap of the cross-fitted predictions. Pairwise AUC differences were estimated in 1,000 stratified bootstrap samples using common out-of-bag patients. The PNI model and ridge model were refitted in each bootstrap sample, with the ridge penalty selected by inner cross-validation; the random forest used the modal configuration selected across the repeated nested-validation folds. Pairwise two-sided empirical P values were calculated from the bootstrap distribution of the AUC difference.

## S5. Random-forest stability analyses and decision curves

The random-forest learning curve used repeated stratified five-fold validation at training fractions of 30%, 50%, 70%, 90%, and 100%. The 500-permutation test refitted the modal nested-validation RF configuration within each five-fold split after outcome-label permutation. Its observed AUC therefore belongs to the fixed-configuration stability pipeline and differs from the primary AUC based on 20 repetitions of nested cross-validation. The empirical permutation P value was calculated as (1 + number of permuted AUCs at least as large as the observed AUC)/(500 + 1). Exploratory decision-curve analysis used averaged out-of-fold probabilities over thresholds 0.10–0.90. Because no clinical decision threshold was prespecified, decision curves were not treated as evidence of clinical utility.

## S6. Cross-fitted SHAP analysis and reproducibility

For each outer validation fold, SHAP values were calculated only for held-out patients using the random forest fitted without those patients. Patient-level SHAP values were averaged across the 20 outer-validation repetitions. Only global mean absolute SHAP importance and the cross-fitted PNI dependence plot were retained. Because the RF train–validation AUC gap was 0.163, exceeding the prespecified 0.10 instability criterion, SHAP was interpreted only as an exploratory description of relative importance and not as evidence of effect direction or causality. Analyses used Python 3.13.9, scikit-learn 1.7.2, SHAP 0.50.0, NumPy 2.3.5, pandas 2.3.3, SciPy 1.16.3, statsmodels 0.14.5, Matplotlib 3.10.6, and seaborn 0.13.2, with global random seed 42.

# Supplementary Tables

Supplementary Table S1. MRI scanners and principal acquisition parameters

| MRI system | | Sequence | | TR/TE (ms) | FOV (mm²) | | Matrix | Slice thickness (mm) | | Interslice gap/overlap (mm) | | DWI b value (s/mm²) | |
| --- | --- | --- | --- | --- | --- | --- | --- | --- | --- | --- | --- | --- | --- |
| MAGNETOM Vida, 3.0 T, Siemens Healthineers | Axial T2WI | | 3570/76 | | 224 × 224 | 320 × 320 | | | 4.0 | | 0.8 gap | | — |
|  | Sagittal T2WI | | 3570/76 | | 224 × 224 | 320 × 320 | | | 4.0 | | 0.8 gap | | — |
|  | Axial DWI | | 5600/46 | | 380 × 306 | 271 × 219 | | | 6.0 | | 1.5 gap | | 1000 |
|  | Axial CE-T1WI | | 3.5/1.66 | | 347 × 347 | 386 × 386 | | | 0.9 | | 0.18 gap | | — |
| Ingenia, 3.0 T, Philips Healthcare | Axial T2WI | | 3276/100 | | 220 × 220 | 292 × 220 | | | 4.0 | | 1.0 gap | | — |
|  | Sagittal T2WI | | 3276/100 | | 220 × 220 | 292 × 220 | | | 4.0 | | 1.0 gap | | — |
|  | Axial DWI | | 6000/56 | | 220 × 220 | 88 × 106 | | | 4.0 | | 1.0 gap | | 1000 |
|  | Axial CE-T1WI | | 3.7/1.33 | | 360 × 320 | 240 × 215 | | | 4.0 | | 2.0 overlap | | — |
| Abbreviations: ADC, apparent diffusion coefficient; CE-T1WI, contrast-enhanced T1-weighted imaging; DWI, diffusion-weighted imaging; FOV, field of view; TE, echo time; TR, repetition time; T2WI, T2-weighted imaging. | | | | | | | | | | | | | |

| Supplementary Table S2 Covariate balance before and after IPTW | | |
| --- | --- | --- |
| Variables | Standardized mean difference before weighting | Standardized mean difference after weighting |
| FIGO stage | 0.296 | 0.006 |
| Pretreatment MRI-assessed LNM | 0.184 | 0.030 |
| Pretreatment maximum tumor diameter on MRI (mm) | 0.180 | -0.024 |
| IPTW, inverse probability of treatment weighting; MRI, magnetic resonance imaging; LNM, lymph node metastasis. | | |

| Supplementary Table S3 IPTW-weighted logistic regression analysis of factors associated with response to neoadjuvant chemotherapy | | | | | |
| --- | --- | --- | --- | --- | --- |
| Variables | β | SE | P value | OR | 95% CI |
| PNI | 0.113 | 0.036 | 0.002 | 1.120 | 1.043–1.202 |
| Pretreatment maximum tumor diameter on MRI (mm) | 0.006 | 0.010 | 0.539 | 1.006 | 0.986–1.027 |
| Constant | -5.790 | 1.961 | 0.003 | — | — |
| IPTW, inverse probability of treatment weighting; PNI, prognostic nutritional index; SE, standard error; OR, odds ratio; CI, confidence interval. | | | | | |

Supplementary Table S4. Cross-fitted performance of the prespecified models and clinical ridge sensitivity model.

A. Discrimination and calibration

| Model | AUC (95% CI) | Brier score | Calibration intercept | Calibration slope |
| --- | --- | --- | --- | --- |
| PNI alone | 0.630 (0.512–0.739) | 0.222 | 0.143 | 0.774 |
| Five-variable ridge LR | 0.684 (0.554–0.798) | 0.210 | 0.159 | 1.312 |
| Random forest | 0.737 (0.624–0.846) | 0.204 | 0.449 | 1.360 |
| Clinical ridge sensitivity | 0.690 (0.567–0.811) | 0.216 | 0.172 | 1.739 |

B. Classification performance at the fixed threshold of 0.5

| Model | Accuracy | Sensitivity | Specificity | PPV | NPV | F1 |
| --- | --- | --- | --- | --- | --- | --- |
| PNI alone | 0.620 | 0.917 | 0.062 | 0.647 | 0.286 | 0.759 |
| Five-variable ridge LR | 0.685 | 0.867 | 0.344 | 0.712 | 0.579 | 0.782 |
| Random forest | 0.696 | 0.750 | 0.594 | 0.776 | 0.559 | 0.763 |
| Clinical ridge sensitivity | 0.707 | 0.850 | 0.438 | 0.739 | 0.609 | 0.791 |

Note: AUC confidence intervals were calculated from stratified bootstrap resampling of patient-level averaged out-of-fold predictions. PPV, positive predictive value; NPV, negative predictive value; PNI, prognostic nutritional index.

|  | | | | | |
| --- | --- | --- | --- | --- | --- |
| Supplementary Table S5. Paired out-of-bag bootstrap comparisons of model AUC. | | | | | |
| Comparison | Mean ΔAUC | Median ΔAUC | 95% CI | *P* | Valid bootstraps |
| Five-variable ridge LR vs PNI | 0.051 | 0.059 | -0.135 to 0.193 | 0.501 | 1000 |
| Random forest vs PNI | 0.082 | 0.085 | -0.125 to 0.260 | 0.378 | 1000 |
| Random forest vs five-variable ridge LR | 0.031 | 0.032 | -0.099 to 0.156 | 0.637 | 1000 |
| Note: Positive ΔAUC favors the first model named in the comparison. Confidence intervals are percentile intervals from 1,000 paired stratified out-of-bag bootstrap repetitions; P values are two-sided empirical values. | | | | | |

| Supplementary Table S6. Association between FIGO stage and pretreatment MRI-assessed LNM and multicollinearity diagnostics for the five-variable prediction specification. | | | |
| --- | --- | --- | --- |
| Panel A： |  |  |  |
| FIGO 2018 stage | MRI-LNM: No | MRI-LNM: Yes | Total |
| IB2–IIA | 21 | 13 | 34 |
| IIB | 4 | 6 | 10 |
| III–IVA | 30 | 18 | 48 |
| Total | 55 | 37 | 92 |
|  |  |  |  |

Panel B：

| Predictor | VIF | Tolerance |
| --- | --- | --- |
| PNI | 1.189 | 0.841 |
| log₂-transformed SCC-Ag | 1.038 | 0.964 |
| log₂-transformed NLR | 1.164 | 0.859 |
| Pretreatment MRI-assessed LNM | 1.029 | 0.972 |
| FIGO: IIB vs IB2–IIA | 1.219 | 0.820 |
| FIGO: III–IVA vs IB2–IIA | 1.237 | 0.809 |
| FIGO stage was dummy encoded with IB2–IIA as the reference category. The five clinical predictors generated six encoded predictor terms because the three-category FIGO variable was represented by two indicator variables. VIF, variance inflation factor; LNM, lymph-node metastasis; PNI, prognostic nutritional index; SCC-Ag, squamous cell carcinoma antigen; NLR, neutrophil-to-lymphocyte ratio. | | |

| Supplementary Table S7. Sensitivity analysis using SCC-Ag and NLR on their original scales in the conventional logistic-regression model. | | | |
| --- | --- | --- | --- |
| Variable | β (SE) | OR (95% CI) | *P* |
| PNI (per unit) | 0.135 (0.061) | 1.145 (1.016–1.291) | 0.027 |
| SCC-Ag (raw) | 0.013 (0.013) | 1.013 (0.987–1.040) | 0.331 |
| NLR (raw) | -0.159 (0.108) | 0.853 (0.690–1.054) | 0.140 |
| LNM (yes vs no) | 0.903 (0.792) | 2.467 (0.523–11.643) | 0.254 |
| FIGO: IIB vs IB2–IIA | -0.566 (0.830) | 0.568 (0.112–2.890) | 0.495 |
| FIGO: III–IV vs IB2–IIA | 0.938 (0.718) | 2.554 (0.625–10.443) | 0.192 |
| Note: The outcome was response=1. IB2–IIA was the reference FIGO category. LNM, lymph node metastasis; NLR, neutrophil-to-lymphocyte ratio; OR, odds ratio; PNI, prognostic nutritional index; SCC-Ag, squamous cell carcinoma antigen. | | | |

| Supplementary Table S8. Random-forest search space and most frequently selected hyperparameters across 100 outer validation folds. | | | |
| --- | --- | --- | --- |
| Hyperparameter | Prespecified candidate values | Most frequent selection | Frequency |
| n_estimators | 300, 800 | 800 | 52/100 (52%) |
| max_features | sqrt, 0.5 | 0.5 | 56/100 (56%) |
| min_samples_leaf | 5, 10, 15 | 5 | 80/100 (80%) |
| min_samples_split | 10, 20, 30 | 10 | 38/100 (38%) |
| max_depth | 3, 5, unrestricted | 3 | 35/100 (35%) |
| class_weight | balanced | balanced | 100/100 (100%) |
| Note: Six reproducibly sampled configurations from the 108-combination prespecified space were evaluated within each outer training fold by four-fold inner cross-validation. The complete fold-level selection record is retained in the reproducibility package. | | | |

| Supplementary Table S9. Exploratory associations between PNI and clinical, laboratory, and pretreatment MRI-derived variables | | | | |
| --- | --- | --- | --- | --- |
| Variable | Coefficient | Coefficient value | P value | FDR-adjusted P value |
| Age | r | 0.078 | 0.462 | 0.874 |
| HPV status | r_rb | 0.412 | 0.172 | 0.552 |
| BMI | ρ | -0.004 | 0.969 | 0.969 |
| Menopausal status | r_pb | 0.073 | 0.492 | 0.874 |
| SCC-Ag | ρ | -0.148 | 0.159 | 0.552 |
| ADC mean | r | -0.033 | 0.754 | 0.949 |
| ADC max | ρ | 0.022 | 0.834 | 0.949 |
| transverse diameter | r | -0.113 | 0.284 | 0.757 |
| anteroposterior diameter | ρ | 0.025 | 0.813 | 0.949 |
| craniocaudal diameter | ρ | -0.023 | 0.829 | 0.949 |
| maximum tumor diameter | ρ | -0.223 | 0.033 | 0.201 |
| tumor necrosis | r_pb | -0.062 | 0.559 | 0.895 |
| tumor differentiation | ρ | -0.015 | 0.89 | 0.949 |
| MRI-assessed LNM | r_pb | 0.077 | 0.468 | 0.874 |
| FIGO stage | ρ | 0.217 | 0.038 | 0.201 |
| Continuous variables were analyzed using Pearson correlation when PNI and the corresponding variable were approximately normally distributed; otherwise, Spearman rank correlation was used. Binary categorical variables were analyzed using point-biserial correlation, except for HPV status, which was evaluated using the Mann–Whitney U test with rank-biserial correlation because the HPV-negative subgroup was small. Patients with unknown HPV status were excluded from the HPV analysis. Ordinal variables were analyzed using Spearman rank correlation. *P* values were adjusted across all 15 tests using the Benjamini–Hochberg false-discovery-rate procedure. | | | | |

# Supplementary Figures

*The random forest met the prespecified instability criterion because the mean train–validation AUC gap was 0.163 (>0.10). Accordingly, Supplementary Figures S3–S4 are descriptive only.*

Supplementary Figure S1. Random-forest learning curve. Training and validation AUCs are shown across training sample sizes corresponding to 30%, 50%, 70%, 90%, and 100% of the available training data under repeated stratified validation. Shaded bands indicate the empirical validation range. The persistent training–validation separation at larger sample sizes supports the instability assessment.

Supplementary Figure S2. Random-forest outcome-permutation test. The histogram shows AUCs from 500 outcome permutations using five-fold validation and the modal RF configuration selected in the repeated nested analysis. The red line denotes the observed fixed-configuration validation AUC of 0.691. The empirical P value was 0.006. This observed AUC differs from the primary repeated nested cross-validation AUC of 0.737 because it belongs to the fixed-configuration stability pipeline.

Supplementary Figure S3. Exploratory cross-fitted random-forest feature importance. Bars show mean absolute SHAP values calculated only in held-out outer validation folds and averaged at the patient level across repeats. Because the random forest was unstable, the ranking is not interpreted as causal or clinically reliable.

Supplementary Figure S4. Exploratory cross-fitted SHAP dependence plot for PNI. The horizontal axis is restricted to the verified observed PNI range of 37.95–58.05. Color represents the three FIGO groups used throughout the analysis: IB2–IIA, IIB, and III–IV. FIGO was coded 1, 2, and 3 only for random-forest splitting and visualization. Directional interpretation is withheld because the random forest was unstable.
